# Secure and Cost-Effective Distributed Aggregation for Mobile Sensor Networks

**DOI:** 10.3390/s16040583

**Published:** 2016-04-23

**Authors:** Kehua Guo, Ping Zhang, Jianhua Ma

**Affiliations:** 1 School of Information Science and Engineering, Central South University, Changsha 410083, China; guokehua@csu.edu.cn (K.G.); zhangp@csu.edu.cn (P.Z.); 2 School of Electronics and Information Engineering, Hunan University of Science and Engineering, 425199 Yongzhou, China; 3 Faculty of Computer and Information Sciences, Hosei University, 184-8584 Tokyo, Japan

**Keywords:** secure data aggregation, mobile sensor networks, approximate aggregation, cost effective, dynamic networks, network security

## Abstract

Secure data aggregation (SDA) schemes are widely used in distributed applications, such as mobile sensor networks, to reduce communication cost, prolong the network life cycle and provide security. However, most SDA are only suited for a single type of statistics (*i.e.*, summation-based or comparison-based statistics) and are not applicable to obtaining multiple statistic results. Most SDA are also inefficient for dynamic networks. This paper presents multi-functional secure data aggregation (MFSDA), in which the mapping step and coding step are introduced to provide value-preserving and order-preserving and, later, to enable arbitrary statistics support in the same query. MFSDA is suited for dynamic networks because these active nodes can be counted directly from aggregation data. The proposed scheme is tolerant to many types of attacks. The network load of the proposed scheme is balanced, and no significant bottleneck exists. The MFSDA includes two versions: MFSDA-I and MFSDA-II. The first one can obtain accurate results, while the second one is a more generalized version that can significantly reduce network traffic at the expense of less accuracy loss.

## 1. Introduction

Wireless sensor networks and mobile sensor networks [1,2,3,4] have received unprecedented attention because of their exciting potential applications in military, industrial and civilian areas (e.g., environmental and habitat monitoring). Wireless communication is often used to transfer data among nodes in these networks, and most nodes are equipped with a battery as the energy unit, which means the energy capacity is limited. Generally, wireless transmission consumes much more energy than data processing. How to save the overall energy resources and extend the lifetime of networks is a popular research topic.

Data aggregation [5,6,7,8,9,10,11,12,13,14] is one of the most important solutions in minimizing the transmitted data size in large-scale wireless networks and is also one of the most important tasks in other distributed applications [15,16,17,18,19,20,21]. Data aggregation can be achieved via *In-server* or *In-network* aggregation. *In-server* aggregation, where data aggregation is performed directly at the server based on the raw data received from each client, is an energy cost approach in large-scale distributed systems. *In-network* aggregation (*i.e.*, aggregating partial results at intermediate nodes along the routing path) significantly reduces the total communication cost and obtains load balance, especially when we only need the aggregation result instead of much raw data.

The data aggregation scheme also faces many security challenges. For example, wireless sensor networks are usually deployed in remote and hostile environments in military applications; thus, sensor nodes are prone to node compromise attacks, and security issues, such as data confidentiality and integrity, are extremely important. Wireless sensors are also being increasingly used to monitor/collect information in healthcare medical systems. It is important to effectively process the ever-growing healthcare data and simultaneously protect patients’ data privacy.

The traditional encryption technology is not suitable for secure data aggregation (SDA) because it only provides concealment and does not support cipher text operations. To realize in-network secure data aggregation based on traditional encryption technology, intermediate aggregators will have to decrypt the data received from all children before operating on them, and then, an encryption will be needed for the aggregated result before sending the message. Frequent encryption and decryption of data in intermediate nodes will increase the computing cost and energy consumption. Moreover, secret key management is more difficult, because the decryption key storage at an intermediate node can be easily obtained by an attacker.

To solve this problem, several secure data aggregation schemes have been proposed, such as synopsis diffusion-based [7,8,9], shuffling-based [6], and homomorphic encryption-based [10,11,12,13,14] data aggregation. Homomorphic encryption-based data aggregation, which has a better theoretical foundation, will be used in the proposed schemes. A comprehensive review on secure data aggregation protocols was presented by Ozdemir *et al.* [22].

Enabling *multi-function* support is also a challenge in the *In-network* data aggregation scheme. Statistics can be divided into two categories according to the aggregation functions used, *i.e.*, *summation-based* and *comparison-based*. For example, aggregation operations, such as median computation or finding the maximum/minimum, rely exclusively on comparison operations. Moreover, aggregation operations, such as count, mean, variance or standard deviation (STD), rely on summation operations. Most of the existing secure data aggregation schemes can only obtain a single type of statistics. To obtain *summation-based* and *comparison-based* statistical results simultaneously is still an open problem.

Take *homomorphic encryption (HE)-based SDA* for example. In [10,11], an additive homomorphism property was used for aggregation on encrypted messages, so that *summation-based* statistics results, such as *CNT (Count)* and *SUM* , can be obtained by these algorithms. However, it could not obtain *comparison-based* statistics, such as *MAX* and *MIN* . Rivest *et al.* [23] noted that any privacy homomorphism is insecure, even against cipher text-only attacks, if it supports comparison operations. Acharya *et al.* [24] applied the order-preserving encryption [25] in SDA to obtain *comparison-based* statistics; however, *summation-based* statistics were not supported in the order-preserving encryption. Ertaul *et al.* [26] and Samanthula *et al.* [27] also only supported *comparison-based* statistics.

Multifunction is also important in other areas. For example, Lu *et al.* [28] presented a multi-function secure data aggregation scheme for smart grid communications. The Boneh–Goh–Nissim cryptosystem was adopted for data privacy, and only summation-based statistics (such as average and variance) were supported in the scheme.

More details regarding the functionality comparison results are listed in Table 1. The second and third columns indicate whether *summation-based* or *comparison-based* statistical results are supported, while the last column indicates whether all statistics can be derived from a single query. “P” means partly supported; “Y” and “N” are “yes” and “no”, respectively. The last row is the proposed scheme. More details regarding Table 1 and the proposed scheme will be given in the following sections.

To enable arbitrary aggregation operations on a *server*, RCDA (Recoverable Concealed Data Aggregation) [12] designed a scheme that can recover all sensing data, even data that have been aggregated. In RCDA, a homomorphic encryption algorithm is used to provide end-to-end confidentiality, and an encode step is used to enable recovery of all sensing data, which means that the scheme can achieve arbitrary method support. However, the final data are formed as concatenations of all sensing data, and no information compression method is used; thus, the communication cost is too heavy.

Nodes do not always remain active in dynamic networks. Some of them may sleep to save energy, and some of them may be dead due to energy exhaustion and other reasons. Most existing SDA schemes are not efficient for dynamic networks due to the varying number of active nodes. For example, [10,11] use extra communication costs to report the number of active (or inactive) nodes in each query, which cost considerable energy. These extra communication costs were even more than those used for sensing data when the percent of active (or inactive) nodes was large enough. The extra traffic increased dramatically along the router path, which easily formed a network bottleneck, reducing the overall network life cycle. More details are listed in Table 2.

In this paper, we propose two *multi-functional secure data aggregation* (MFSDA) schemes, *i.e.*, *MFSDA-I* and *MFSDA-II*. Both of them can obtain addition-based and comparison-based statistics at the same query. The first one can obtain accurate results, while the second one is an approximate version that can significantly reduce communication cost and prolong the network life cycle at the expense of less accuracy loss. More specifically, to provide *value-preserving* and *order-preserving* during in-network aggregation and then enabling arbitrary statistics support, we introduce a *mapping* step and a *coding* step in the proposed scheme. A *compressing* step is introduced to further reduce the packet size in *MFSDA-I*.

The remaining part of this paper is organized as follows: Section 2 introduces the network model and the background knowledge. Section 3 and Section 4 introduce MFSDA-I and MFSDA-II, respectively. Section 5 presents functionality and security analysis. Section 6 presents performance analysis and evaluation. Sections 7 and 8 offer a summary and acknowledgment.

## 2. Preliminaries

In this section, we first introduce the network model and attack model. Then, we give the encryption and signature schemes used in the proposed schemes. The final subsection lists the basic notations.

### 2.1. Network Model

**Figure 1:**
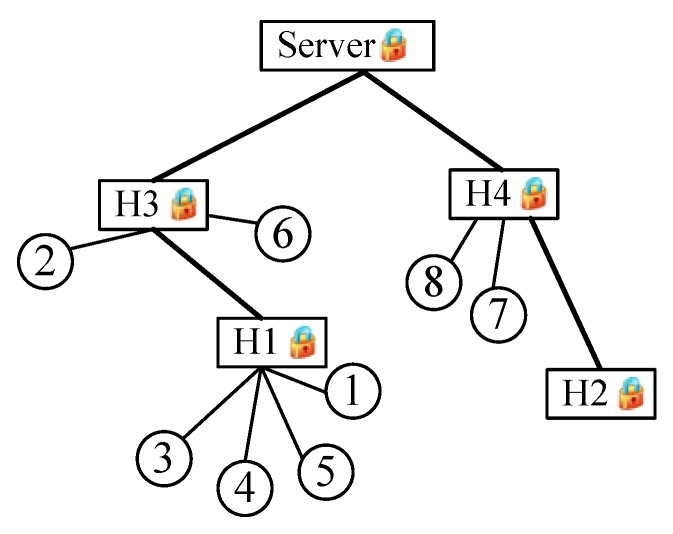
Network model.

As shown in Figure 1, a cluster-based topology is used. It is composed of a *server* and a large number of *clients/nodes*. The nodes, selected as cluster heads (*CH*, e.g., *H1*, *H2*, *etc.*), are assumed to be trustworthy, which means that secret information can be stored if required. The remaining nodes (*CM*, cluster member, e.g., 1, 2, *etc.*) choose appropriate clusters to add themselves to according a certain criterion, such as signal strength in a wireless network and delay in a wired network. All *CHs* form a tree, with the *server* as its root. For the convenience of analysis, assume that only cluster members generate data.

### 2.2. Attack Model

Assume that the adversary is rational; which means that he or she will never expose himself or herself to obtain information; malicious destruction of nodes will never happen.

Adversaries know all public keys and other public parameters. The private data stored in *CHs* will have been destroyed before the adversary captures it.

#### 2.2.1. Without Compromising any *Nodes*

If an adversary does not compromise any *nodes*, the adversary can still launch an attack in wired/wireless channel. Let us consider the following situations.

**A1:**  **Eavesdrop**: The adversary can eavesdrop on any data transmission in a wired/wireless channel.
**A2:**  **Replay attack**: An adversary can use historical data packets instead of an actual data packet, to interfere with the normal information acquisition.
**A3:**  **Data tampering**: An adversary can modify the data packet and send to any nodes.
**A4:**  **DoS Attack**: The forge massage will easily spread in end-to-end data aggregation technology due to the multi-hop flooding effect. When an adversary injects a forged message, the final aggregation result the *server* got is always wrong, thus forming a DoS attack.

#### 2.2.2. Compromising *CM*

If an adversary has compromised one or more *CM*, it can obtain all of its secrets. An adversary can use this private information and other public information to modify or forge packets.

Generally, the local value of an honest node is bounded, and then, the compromised node can falsify its own sensor reading as follows:

**B1:**  A compromised node falsifies the local value outside the bound.
**B2:**  A compromised node falsifies the local value within the bound.

### 2.3. Encryption and Signature Scheme

Because elliptic curve cryptography (ECC) can reduce key size with high security, it provides us with calculating speed and computation cost. Both the encryption and signature scheme used here are ECC-based.

#### 2.3.1. Homomorphic Encryption Scheme

The homomorphic encryption (HE) scheme is derived from homomorphism in abstract algebra. Using homomorphism, operations in one algebraic system (plaintext) can be mapped into an operation in another algebraic system (cipher text). The HE scheme has been widely applied in secure data aggregation [11,12,13,31].

An asymmetric HE scheme (Algorithm A1 in Appendix A), which is derived from the ElGamal encryption scheme (EC-EG) [32], will be used in MFSDA. Details are illustrated in Appendix A.

Based on the homomorphic property described in Theorem 1, the intermediate nodes perform data aggregation directly in cipher text. Without frequent encryption and decryption, the computation overhead is reduced, and the key management is easy.

**Theorem 1.**  *(Homomorphic property) Algorithm A1 has an additive homomorphic property, namely the summation arithmetic in plaintext is equivalent to summation arithmetic in cipher text,* *i.e.*,

$$HEnc\left(m_{1}+m_{2}\right)=HEnc\left(m_{1}\right)⨁HEnc\left(m_{2}\right)$$

The proof refers to Appendix A.

#### 2.3.2. Identity-Based Signature Scheme

In the identity-based signature (IBS) scheme, users’ public identity information (ID, email, *etc.*) is used as the public key for signature verification, which can effectively solve the problems in the management of PKI public-key certificates.

The algorithm, which is derived from [33], consists of four parts: setup, extract, signature and verify. Details are illustrated in Appendix B.

### 2.4. Basic Notation

Table 3 lists the notations that we will use later.

## 3. Multi-Functional Secure Data Aggregation Scheme: MFSDA-I

Two *multi-functional secure data Aggregation* schemes are proposed: *MFSDA-I* and *MFSDA-II*. *MFSDA-I* is given in this section, while *MFSDA-II* will be given in the next.

### 3.1. MFSDA-I

*MFSDA-I* consists of four parts (procedures): setup and operations on the three types of nodes, *i.e.*, *cluster member (CM,* e.g., *1, 2, etc.)*, *cluster head (CH,* e.g., *H1, H2, etc.)* and *server*.

The setup procedure includes network initialization, as well as the initialization of encryption and signature. Both non-homomorphic and homomorphic encryption are used in the proposed scheme. The former is used in intra-cluster data transmission; the latter is used in inter-cluster data transmission. Intra-cluster encryption is the same as the one used in RCDA [12]. For Details regarding inter-cluster encryption, refer to Section 2.3.1. An IBS signature mechanism (see Section 2.3.2) is used for all packets.

Each *CM* encrypts the raw data with the non-homomorphic encryption mentioned above. Then, a timestamp and other information are attached to the cipher text before IBS signature application. The final packets sent to the *cluster head (CH)* consist of all of them.

Each *CH* first verifies the signature of all of the received packets. The received packets can be divided into two categories: one is intra-cluster data, which are received from the *CM* node within a cluster, and inter-cluster data, which are from other cluster heads.

For the intra-cluster data, the *CH* first decrypts the data and then maps and encodes it to obtain a vector. All vectors generated in the same cluster are added up in plaintext to obtain an intra-cluster data aggregation result. The aggregated vector will be encrypted using the homomorphic encryption scheme with the public key of the server.

For the inter-cluster data, because they have been encrypted using the homomorphic encryption scheme in other *CH* nodes, there is no need to also decrypt it. The *CH* will sum two types of cipher text above directly in the cipher text domain. The final packet sent to the parent node (cluster head or server) also contains a time stamp, other information and the signature generated from them using IBS with the private key of the cluster head.

All packets received by the *server* are from cluster heads. The *server* first verifies them, then extracts the encrypted data and aggregates them in the cipher domain. Finally, the *server* decrypts it to obtain the aggregation result of the whole network.

We can extract all common statistics from the aggregation result, which is in a vector form, using the functions provided in following section.

#### 3.1.1. Setup

The setup procedure initializes the network topological structure, as well as initializes each encryption and signature mechanism. The following key pairs will be generated during the setup.

##### 1. $<ID_{k},KeyLH_{k}>$:

This type of key pair, which was used in RCDA [12], is used for intra-cluster data encryption, namely data transmitted between *CM* and its *CH*.

In a large-scale distributed system, the *server* does not know the cluster information before deployment, and the key preload scheme is infeasible. A key exchange scheme [12] is introduced to solve this challenge.

Each *CM* loads its own key pair, generated by the *server*. All *CH* will generate a span tree, with the *server* as its root. Each *CM* joins a suitable cluster based on a certain criterion (e.g., delay in a wired network and signal strength in a wireless network), encrypts the cluster choice with its private key and transmits it to the *server*. After decryption, the *server* will send the *CM*’s key pairs to its *CH*. Thus, the CH can obtain all of the key pairs of its *CM*s.

##### 2. $<ID_{k},KeySign_{k}>$:

This type of key pair is used for the IBS signature mechanism (Section 2.3.2). To ensure the integrity of the data, all packets, including intra-cluster and inter-cluster, must be attached with a signature.

The identifier $ID_{k}$ of each node is also the public key for signature verification. The private key $KeySign_{k}$ of each node is generated by the *server* using the master key. This key pair is preloaded into each node before deployment. Each packet contains a public key and a signature generated by the sender using its private key. The receiver will verify all of the packets before further processing.

##### 3. <KeyPriS,KeyPubS>:

This key pair is used for homomorphic encryption (Section 2.3.1) to achieve inter-cluster data confidential, namely the data transmission between *CH*s or between the *CH* and the server. The public key of the *server* (*i.e.*, KeyPubS) is used for data encryption at the *CH*. The *server* keeps the private key KeyPriS for decryption. In the proposed scheme, all of the data contained in the inter-cluster packets are homomorphism encrypted, so data aggregation is performed directly without decryption.

#### 3.1.2. Operations on *CM*

Operations on the *CM* are composed of three parts: *encryption*, *signature* and *data transmission*.

First, each *CM* encrypts the sensing data $x_{k}$ with the encryption key $KeyLH_{k}$ to obtain $C_{k}$. Then, using the private key $KeySign_{k}$, the *CM* generates the signature from the transmitting data Msg, which consists of ciphertext, a timestamp and other information. Finally, the *CM* transmits Msg and $S_{k}$ to the *CH*.

1. **Encryption**: $C_{k}=encrypt\left(x_{k},KeyLH_{k}\right)$.
2. **Signature**:
  $$\begin{array}{c} \;\;\;\;S_{k}=signature\left(Msg,KeySign_{k}\right)\\ Msg=\{IDsrc,IDdest,timestamp,msgType=tLH,C_{k}\} \end{array}$$

IDsrc and IDdest are the identification (ID) of the *CM* and its cluster head (*CH*), respectively. A timestamp is adopted to avoid message duplication and to process data by intervals. In addition, if an adversary attempts to modify the timestamp without a valid private key for the signature, the receiver can detect it by a signature verification mechanism. There are two types of messages in the scheme, defined as msgType: one for intra-cluster communication, denoted as tLH; another is used for inter-cluster communication, denoted as tHH. Here, the message type is the former, namely msgType=tLH.

3.  **Messages Send**:

The final data packet sent by *CM* to its *CH* is

$$MsgSend=\{Msg,S_{k}\}$$

#### 3.1.3. Operations on *CH*

Operations on the *CH* consist of five parts: *data receiving and verification*, *classification*, *operations on the intra-cluster dataset*, *operations on inter-cluster dataset* and *packet construction and transmission*.

The *CH* will verify all received message MsgRec and eliminate the one that fails. The verification process includes: check the legality of IDsrc and IDdest; check the effectiveness of the timestamp; and verify the signature.

Legitimate messages will be divided into two categories, *i.e.*, {intraSet} and {interSet}, according to the msgType. {intraSet} contains the intra-cluster packets received from each *CM* in the cluster. {interSet} contains the inter-cluster packets received from other *CH* as its children.

The process for the intra-cluster dataset {intraSet} consists of five steps: decryption, mapping, encoding, aggregation and re-encryption. “Mapping, encoding and aggregation”, which will be further explained in the following section, are the key to ensuring the simultaneous extraction of various types of statistics. The public key of the *server* (*i.e.*, KeyPubS) is used in homomorphic encryption.

All of the messages contained in {interSet} are encrypted by the public key of the server. The data aggregation of the dataset can be performed directly in the encrypted domain due to the homomorphic attribution of the encryption mechanism.

Messages in {intraSet} and {interSet}, have been transformed into CAggIntra and CAggInter at previous two steps. The *CH* firstly sums them up in the cipher text domain and attaches other information, such as the timestamp. Then, the *CH* generates a signature $S_{k}$ using the private key. Finally, the *CH* sends Msg and $S_{k}$ to the parent node.

1. **Data receiving and verification**:
  {MsgRec}={MsgSend}
  $$verifiy(MsgRec,ID_{k})$$
2. **Classification**:
  {intraSet}={MsgRec|msgType==tLH}
  {interSet}={MsgRec|msgType==tHH}
3. **Operations on {intraSet}**
  (1) **Decryption**: $x_{k}=decrypt\left(C_{k},keyLH_{k}\right)$
  (2) **Mapping**: $y_{k}=f_{m}\left(x_{k}\right)$
  (3) **Encoding**: ${\vec{v}}_{k}=f_{e}\left(y_{k}\right),\;\;\;$ while ${\vec{v}}_{k}\in {\left\{0,1\right\}}^{L}$
  (4) **Aggregation**: ${\vec{V}}_{j}=\sum_{k=1}^{N_{j}}{\vec{v}}_{k}$
  (5) **Encryption**:
    $$CAggIntra=HEnc({\vec{V}}_{j},KeyPubS)$$
4. **Operations on {interSet}**:
  $$CAggInter=\sum_{msg_{i}\in \left\{interSet\right\}}C_{i}$$
5. **Signature and Send**
  (1) **Aggregation**:
    $$C_{i}=CAggIntra+CAggInter$$
  (2) **Signature**:
    $$\begin{array}{c} \;\;\;\;S_{i}=signature\left(Msg,KeySign_{i}\right)\\ Msg=\{IDsrc,IDdest,timestamp,msgType=tHH,C_{i}\} \end{array}$$
  (3) **Messages send**:
    The packet sent to the parent is:
    $$MsgSend=\{Msg,S_{i}\}$$

#### 3.1.4. Operations on the Server

To simplify the analysis, all of the children of the *server* are assumed to be the *CH*.

Operations on the *server* consist of four parts: *data receiving and verification*, *aggregation*, *decryption* and *statistical results acquisition*.

1. **Data receiving and verification**:
  Message receiving and verification is the same as that of the *CH*.
2. **Aggregation**:
  $$C_{S}=\sum_{msg_{i}\in \left\{interSet\right\}}C_{i}$$
3. **Decryption**:
  $$\vec{V}=HDec\left(C_{S},keyPriS\right)$$
4. **Get the statistic result**:
  Each statistic result can be obtained directly from $\vec{V}$ using the following formulas.
  **CNT**: $CNT=\sum_{i=1}^{L}n_{i}$
  **SUM**:
    $$SUM\left(x\right)=\sum_{k=1}^{N}x_{k}=\sum_{k=1}^{N}f_{m}^{-1}\left(y_{k}\right)=\sum_{i=1}^{L}f_{m}^{-1}\left(i\right)\times n_{i}$$
  **MEAN**: $MEAN=\frac{SUM(x)}{CNT}$
  **VAR**:$VAR=E\left(x^{2}\right)-E{\left(x\right)}^{2}$
  where
  $$\begin{array}{c} E(x)=MEAN\;\\ E\left(x^{2}\right)=\frac{SUM(x^{2})}{CNT}\;\\ SUM\left(x^{2}\right)=\sum_{k=1}^{N}x_{k}^{2}=\sum_{k=1}^{N}{\left(f_{m}^{-1}(y_{k})\right)}^{2}=\sum_{i=1}^{L}{\left(f_{m}^{-1}\left(i\right)\right)}^{2}\times n_{i} \end{array}$$
  **STD**: $STD=\sqrt{VAR(x)}$
  **MAX**: $MAX=f_{m}^{-1}\left(i_{\max}\right)$
  where
  $$\begin{array}{c} i_{\max}=\max\left\{i|i\in \left(0,L\right]\;\&\&\;n_{i}>0\right\} \end{array}$$
  **MIN**: $MIN=f_{m}^{-1}\left(i_{\min}\right)$
  where
  $$\begin{array}{c} i_{\min}=\min\left\{i|i\in \left(0,L\right]\;\&\&\;n_{i}>0\right\} \end{array}$$

### 3.2. Mapping, Encoding and Aggregation

In this subsection, we first present details regarding mapping and encoding and then introduce the aggregation step.

#### 3.2.1. Details of Mapping and Encoding

As mentioned above, statistics results can be divided into two types: addition-based statistics (such as SUM, AVG, VAR, *etc.*) and comparison-based statistics (such as MAX, MIN, *etc.*). The former requires *value-preserving*, while the latter requires *order-preserving*.

Obtaining the statistical results of many types effectively using only one query is difficult because it is hard to keep *value-preserving* and *order-preserving* attributions simultaneously during the aggregation of encrypted data.

In the proposed scheme, we can maintain the two types of information mentioned previously in the secure data aggregation process by using *mapping* and *encoding*.

**Figure 2:**
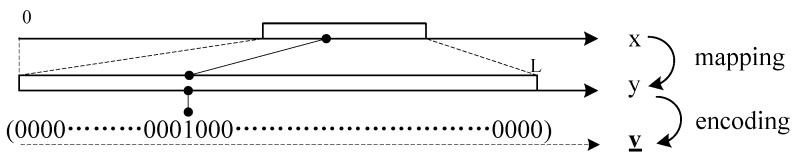
Mapping and encoding step of MFSDA-I.

As shown in Figure 2, there are three types of data in the proposed scheme: sensing data *x*, mapped data *y* and encoded data $\vec{v}$. Mutual conversion can be performed through the mapping, encoding and inversion of them.

Sensing data $x_{k}$ are the original data gathered at node *k*. $x_{k}$ belong to a subset of real domain, *i.e.*, $x_{k}\in \left(X_{LB},X_{UB}\right]$, where $X_{LB}$ and $X_{UB}$ are the lower and upper bounds, respectively.

$x_{k}$ is transformed into mapped data $y_{k}$ using the *mapping* step. $y_{k}$ belong to a subset of the natural numbers, *i.e.*, $y_{k}\in \left(0,L\right]$, where $L=\lceil \frac{X_{UB}-X_{LB}}{a}\rceil$, and *a* is the accuracy requirement of $x_{k}$.

The conversion between $x_{k}$ and $y_{k}$ is achieve by the *mapping* function and its inverse, *i.e.*, $f_{m}$ and $f_{m}^{-1}$.

$$\begin{array}{c} y_{k}=f_{m}\left(x_{k}\right)=\frac{x_{k}-X_{LB}}{a}\\ x_{k}=f_{m}^{-1}\left(y_{k}\right)=a\times y_{k}+X_{LB} \end{array}$$

$y_{k}$ is converted into ${\vec{v}}_{k}$ by *encoding*. ${\vec{v}}_{k}$ is a vector, ${\vec{v}}_{k}\in {\left\{0,1\right\}}^{L}$; its elements’ number is *L*. The element of $\left(y_{k}\right)$-th is one; other elements are zero; that is to say:

$${\vec{v}}_{k}\left(i\right)=\left\{\begin{array}{c} \begin{array}{cc} 1 & \left(i=y_{k}\right) \end{array}\\ \begin{array}{cc} 0 & \left(i\neq y_{k}\right) \end{array} \end{array}\right.$$

The conversion between $y_{k}$ and ${\vec{v}}_{k}$ is achieve by the encoding function and its inverse, *i.e.*, $f_{e}$ and $f_{e}^{-1}$.

$$\begin{array}{c} {\vec{v}}_{k}=f_{e}\left(y_{k}\right)\\ y_{k}=f_{e}^{-1}\left({\vec{v}}_{k}\right) \end{array}$$

#### 3.2.2. Aggregation

Aggregation is performed on the plaintext or cipher text of ${\vec{v}}_{k}$. An example for the former is the 3-(4)th step in Section 3.1.3; an example for the latter is the fourth step in Section 3.1.3 and the second step in Section 3.1.4. The following theorem proves that the *server* could finally obtain the aggregated result of all sensing data of active nodes.

**Theorem 2**: *The homomorphic encryption used in the proposed scheme can ensure that the vector obtained at the server after decryption is the aggregated result of all encoded data of active nodes.*

For the proof, refer to Appendix C

Despite that the elements of ${\vec{v}}_{k}$ can only be zero or one, but usually the vector elements of the final aggregated, result $\vec{V}=\sum {\vec{v}}_{k}$ may be arbitrary natural numbers that are not greater than N.

### 3.3. A Concrete Example

Figure 1 is the network model of this example. Assume that $x_{k}\in \left(20,25\right]$ and that the accuracy requirement is a=1. Sensing data gathered from each node are listed in the second column in Table 4. The third and fourth columns present mapped data $y_{k}$ and encoded data ${\vec{v}}_{k}$, respectively.

Each *CM* encrypts the sensing data, attaches the signature and other information and transfers these to the cluster head (*CH*).

The *CH* verifies the received message and obtains the sensing data via decryption. Mapped data $y_{k}$ ($y_{k}\in \left(0,5\right]$) are transformed from $x_{k}$ using the mapping function.

Because the sensing data of Nodes 3 and 5 are outside of the valid data range (20,25], the *CH* node will regard them as illegal data and discard them.

Other valid mapped data are encoded as a vector ${\vec{v}}_{k}$ whose length is L. The $y_{k}$-th element is one, while all of the remaining elements are set to zero. Let us take Node 1 as an example; the sensing data are $x_{1}=23$; mapped data $y_{k}=3$ are obtained after the mapping step; and then, the third element of the vector is set to one; while other elements are zero, *i.e.*, ${\vec{v}}_{k}=\left(0\;0\;1\;0\;0\right)$. The values of each step are listed in Table 2.

For all of the intra-cluster data, the *CH* encodes them first, then sums and encrypts the summation using the *server* public key of homomorphic encryption. For all of the inter-cluster data, the CH adds them up directly in the cipher text domain after verification. Both the intra-cluster and inter-cluster data are homomorphic encrypted data in the *server* public key; thus, they can be summed up directly in the cipher text domain, which is equivalent to getting the cipher text of the regional data aggregation result. Finally, this regional result is transmitted to the parents, attached with other information, such as time stamp, signature, *etc.*

According to the homomorphic property, the summation arithmetic of vectors in the cipher text domain is equivalent to that in plaintext. Therefore, the *server* can obtain the final aggregation result $\vec{V}=\sum {\vec{v}}_{k}$ by decrypting the received data. For example, in this case, the final data the *server* obtains are the summation of the vector in the fourth column of Table 4.

$$\vec{V}=\sum {\vec{v}}_{k}=\left(0\;1\;3\;1\;1\right)$$

Each statistic can be calculated directly from $\vec{V}$.

$$\begin{array}{c} CNT=\sum_{i=1}^{L=5}n_{i}=6\\ SUM\left(x\right)=\sum_{i=1}^{L=5}\left(i+20\right)\times n_{i}\\ \;\;\;\;\;\;\;\;\;\;\;\;=21\times 0+22\times 1+23\times 3+24\times 1+25\times 1=140\\ MEAN=SUM(x)/CNT=140/6\approx 23.33\\ i_{max}=\max\left(\left\{i|i\in \left(0,L\right]\&\&n_{i}>0\right\}\right)=5\\ i_{min}=\min\left(\left\{i|i\in \left(0,L\right]\&\&n_{i}>0\right\}\right)=2\\ Max=f_{m}^{-1}\left(i_{max}\right)=25\\ Min=f_{m}^{-1}\left(i_{max}\right)=22 \end{array}$$

$$\begin{array}{c} SUM\left(x^{2}\right)=\sum_{i=1}^{L=5}{\left(i+30\right)}^{2}\times n_{i}\\ \;\;\;\;=21^{2}\times 0+22^{2}\times 1+23^{2}\times 3+24^{2}\times 1+25^{2}\times 1\\ \;\;\;\;=3272\\ E(x)=MEAN\\ E\left(x^{2}\right)=SUM\left(x^{2}\right)/CNT=545.3333\\ VAR=E\left(x^{2}\right)-E{\left(x\right)}^{2}\approx 0.89\\ STD=\sqrt{VAR}\approx 0.94 \end{array}$$

## 4. Multi-Functional Secure Data Aggregation Scheme: MFSDA-II

On the one hand, we can make a decision without accurate statistical results in most WSN applications; on the other hand, we can acquire performance improvement (such as reducing the amount of the reduction of energy consumption, communication, *etc.*) by reducing the accuracy requirement. Therefore, a large number of approximation algorithms have been proposed [7,8,9,29,34,35,36].

According to the analysis above, we know that the total data transmission is still large while L is large. Thus, we propose an approximation scheme in which a data compression is introduced to reduce the total data transmission and prolong the network life cycle.

### 4.1. MFSDA-II

To reduce the communication cost, a compression step is introduced after the mapping step in MFSDA-II. Mapping data *y* are compressed from a larger space with a size of *L* into a smaller space with a size of $L^{\prime }$. The encoding step is executed on compression data *z*, which makes the vector length decrease from *L* to $L^{\prime }$.

**Mapping**: The mapping step is the same as the one in MFSDA-I. The mapping function and its inverse function are as follows:

$$\begin{array}{c} y_{k}=f_{m}\left(x_{k}\right)\\ x_{k}=f_{m}^{-1}\left(y_{k}\right) \end{array}$$

**Compressing**: The compression function $f_{c}$ will convert $y_{k}$ into $z_{k}$ as follows, where *c* is the compression factor.

$$z_{k}=f_{c}\left(y_{k}\right)=\left\lceil \frac{y_{k}}{c}\right\rceil =\left\lceil \frac{f_{m}\left(x_{k}\right)}{c}\right\rceil$$

One can recover ${\hat{y}}_{k}$ as an estimate of $y_{k}$, using the following decompressing function on $z_{k}$:

$${\hat{y}}_{k}=f_{c}^{-1}\left(z_{k}\right)=c\times z_{k}-\left\lfloor \frac{c}{2}\right\rfloor$$

**Encoding**: The encoding step is based on $z_{k}$ instead of $y_{k}$, *i.e.*, ${\vec{v}}_{k}=f_{e}\left(z_{k}\right)$, while ${\vec{v}}_{k}\in {\left\{0,1\right\}}^{L^{\prime }}$, and:

$${\vec{v}}_{k}\left(i\right)=\left\{\begin{array}{c} \begin{array}{cc} 1 & \left(i=z_{k}\right) \end{array}\\ \begin{array}{cc} 0 & \left(i\neq z_{k}\right) \end{array} \end{array}\right.$$

We can recover ${\vec{v}}_{k}$ from ${\hat{y}}_{k}$, for that $z_{k}$ and ${\vec{v}}_{k}$ are equivalent to each other.

$${\hat{y}}_{k}=f_{c}^{-1}\left(z_{k}\right)=f_{c}^{-1}\left(f_{e}^{-1}\left({\vec{v}}_{k}\right)\right)=f_{c}^{-1}\left(i\right)=c\times i-\left\lfloor \frac{c}{2}\right\rfloor$$

where *i* is the subscript of nonzero elements in ${\vec{v}}_{k}$.

**Aggregation**:

$$\vec{V}=\sum_{k=1}^{N}{\vec{v}}_{k}$$

### 4.2. Get the Statistical Result

The aggregation vector of all active nodes obtained at the *server* is $\vec{V}=\left(n_{1}\;n_{2}\;.\;.\;n_{L^{\prime }}\right)$. Then, we can recover all statistics results from $\vec{V}$ using the following formulas.

**Count (CNT)**: $CNT=\sum_{i=1}^{L^{\prime }}n_{i}$
**Summation (SUM)**: $SUM\left(\hat{x}\right)=\sum_{k=1}^{N}{\hat{x}}_{k}=\sum_{k=1}^{N}f_{m}^{-1}\left({\hat{y}}_{k}\right)=\sum_{k=1}^{N}f_{m}^{-1}(f_{c}^{-1}\left(z_{k}\right))=\sum_{i=1}^{L^{\prime }}n_{i}f_{m}^{-1}\left(f_{c}^{-1}\left(i\right)\right)$
**Average/Mean (MEAN)**: $MEAN=\frac{SUM(\hat{x})}{CNT}$
**Variance (VAR)**: $VAR\left(\hat{x}\right)=E\left({\hat{x}}^{2}\right)-E{\left(\hat{x}\right)}^{2}$

where

$$\begin{array}{c} E\left(\hat{x}\right)=MEAN;E\left({\hat{x}}^{2}\right)=SUM\left({\hat{x}}^{2}\right)/CNT;\\ SUM\left({\hat{x}}^{2}\right)=\sum_{k=1}^{N}{\hat{x}}_{k}^{2}=\sum_{k=1}^{N}{\left(f_{m}^{-1}\left({\hat{y}}_{k}\right)\right)}^{2}\\ =\sum_{k=1}^{N}f_{m}^{-1}(f_{c}^{-1}\left(z_{k}\right){)}^{2}=\sum_{i=1}^{L^{\prime }}n_{i}{\left(f_{m}^{-1}\left(f_{c}^{-1}\left(i\right)\right)\right)}^{2} \end{array}$$

**Standard Deviation (STD)**: $STD=\sqrt{VAR(\hat{x})}$
**Maximum (MAX)**: $MAX=f_{m}^{-1}\left(f_{c}^{-1}\left(i_{\max}\right)\right)$

where:

$$\begin{array}{ccc} & & i_{\max}=\max\left\{i:i\in \left(0,L^{\prime }\right]\;\&\&\;n_{i}>0\right\} \end{array}$$

**Minimum (MIN)**: $MIN=f_{m}^{-1}\left(f_{c}^{-1}\left(i_{\min}\right)\right)$

where:

$$\begin{array}{ccc} & & i_{\min}=\min\left\{i:i\in \left(0,L^{\prime }\right]\;\&\&\;n_{i}>0\right\} \end{array}$$

### 4.3. An Example of MFSDA-II

Let us illustrate the variant scheme with an example. Assume that the range of sensor data $x_{k}$ is $x_{k}\in \left(9.0,11.0\right]$. After error data are detected and eliminated, seven effective perception data are left, *i.e.*,

$$x_{k}{|}_{k=1}^{7}=\left\{9.7,9.9,9.3,9.4,10.2,10.5,10.9\right\}$$

Mapped data $y_{k}\in \left(0,20\right]$ can be obtained by using the mapping function as follows.

$$y_{k}=f_{m}\left(x_{k}\right)=\left(x_{k}-9\right)\times 10$$

Additionally, the corresponding mapped data are:

$$y_{k}{{|}_{k=1}^{7}=f_{m}\left(x_{k}\right)|}_{k=1}^{7}=\left\{7,9,3,4,12,15,19\right\}$$

$${\vec{V}}_{0}=\sum {\vec{v}}_{k0}=\left(0\;0\;1\;1\;0\;0\;1\;0\;1\;0\;0\;1\;0\;0\;1\;0\;0\;0\;1\;0\right)$$

In MFSDA-II, mapped data have been compressed into compressed data $z_{k}\in \left(0,5\right]$ before the encoding step, *i.e.*,

$$z_{k}{|}_{k=1}^{7}=\left\lceil \frac{f_{m}\left(x_{k}\right)}{c}\right\rceil ={\left\{2,3,1,1,3,4,5\right\}}_{c=4}$$

The encoding step is based on compressed data, so the vector length is reduced from 20 to five. The final aggregation vector is:

$$\vec{V}=\sum {\vec{v}}_{k}=\left(2\;1\;2\;1\;1\right)$$

The total communication cost is only $\frac{1}{4}$ of that in MFSDA-I. The transmission reduction is achieved via loss compression; thus, error exists. The final errors of each statistic require further analysis due to the positive and negative errors existing simultaneously.

We can obtain the common statistics from the final aggregated vector using the formula above. For the detailed calculation processes, please refer to Appendix D.

## 5. Functionality and Security Analysis

In this section, we will analyze the functionality and security of the proposed scheme and compare it to other conventional schemes. More specially, the properties, such as *multi-function support*, *dynamic network adaption* and *security*, will be discussed.

### 5.1. Functionality Comparison

The functionality comparison results are listed in Table 1. The second column indicates whether summation-based statistics are supported, while the third indicates whether a comparison-based statistics are supported. The last column indicates whether all statistics can be derived from a single query.

Most of these secure data aggregation (SDA) schemes, such as [9,11], only supported addition-based statistics. Acharya *et al.* [24] only supported comparison-based statistical results. In all of these SDA schemes, different statistics are derived from different queries.

Moreover, even a single statistic may also require several different queries. For example, to obtain VAR in CDA [11], we need at least one CNT query and two SUM queries, *i.e.*, SUM(x) and $SUM(x^{2})$, and obtain VAR by $\frac{SUM(x^{2})}{CNT}-{\left(\frac{SUM(x)}{CNT}\right)}^{2}$.

Both RCDA and the proposed scheme (MFSDA) can obtain all of the common statistical results simultaneously in a single query. However, RCDA has several flaws [13,31] with respect to security and communication costs. A solution for the security weaknesses of RCDA has been discussed in Sen-SDA [31]. The main contribution of Sen-SDA is to improve the efficiency of multiple signature verifications, which is not the same as in this paper; thus, we do not choose it as a candidate for comparison. EERCDA (Energy Efficient Recoverable Concealed Data Aggregation) [13] uses a *differential data transfer method* to reduce the energy consumption in RCDA. For further comparisons, refer to Section 6.

### 5.2. Dynamic Networks Adaptive

Nodes do not always remain active in dynamic networks [8]. Most existing SDA schemes are not efficient for dynamic networks due to the varying number of active nodes. To report abnormal nodes, extra communication costs were used in [10,11], which required much energy and easily formed a network bottleneck. These extra communication costs were even more than those used for sensing data when the percent of active (or inactive) nodes was large enough.

More details regarding comparisons are listed in Table 2. The second column indicates that dynamic networks are not supported, while the third and fourth columns indicate that dynamic networks can be supported with or without extra communication costs, respectively.

### 5.3. Security Analysis

*Security* is one of the most important properties of the secure data aggregation (SDA) scheme. In this subsection, we will analyze the security of MFSDA and compare it to other well-known SDA schemes.

The comparisons results are listed in Table 5. The attack models used in the table header are defined as follows.

#### 1. Without compromising any nodes:

**A1:**  **Eavesdrop**. The privacy of data is not affected by passive monitoring because sensing data have been encrypted in this scheme.
**A2:**  **Replay attack**. The lifetime of each packet is marked by a timestamp in the proposed scheme. If an adversary attempts to modify the timestamp without a valid private key for the signature, the receiver can detect it via the signature verification mechanism.
**A3:**  **Data tampering**. Data tampering can be detected by using the signature mechanism.
**A4:**  **DoS attack**. The parent node can find the illegal data in time; thus, the multi-hop flooding effect hardly spreads.

#### 2. Compromising *CM*:

If an adversary has compromised one or more *CM*, it can obtain all of their secrets. An adversary can use this private information and other public information to modify or forge packets. Generally, the local value of an honest node is bounded, and then, the adversary can falsify the sensor reading of the compromised node as follows:

**B1:**  A compromised node falsifies the local value outside the bound.
**B2:**  A compromised node falsifies the local value within the bound.

## 6. Performance Analysis and Evaluation

In this section, we will analyze the performance of the two schemes proposed in this paper, namely MFSDA-I and MFSDA-II. The former provides an exact result, while the latter can significantly reduce network traffic at the expense of less accuracy loss.

### 6.1. Evaluation Settings

A cluster-based network model is used here. Cluster heads are selected in advance and assumed to be trusted nodes. For smaller scale networks, cluster heads can communicate directly with the server. For a relatively large-scale network, all cluster heads form a tree with the server as its root.

Since network construction and maintenance is not the research focus of this article. We have tried to weaken the impact of the problem with reasonable simplification. In the proposed schemes, the cluster head can be pre-selected. Because of their small numbers, they can be equipped with more batteries. Therefore, these cluster head can be kept active during the lifetime of the WSN or kept active within a predetermined period for data receiving.

To simplify the analysis, the position of each cluster head is assumed to never change, and the tree structure among these cluster heads is assumed to be predetermined. This is feasible due to the limited number of cluster heads. Each cluster head broadcasts its own cluster formation requests periodically.

The interval of these cluster formation requests can be predefined based on the possible maximum move speed of the network nodes. For example, if the position of the node changes slowly or never changes, then it is necessary to enlarge this interval to reduce energy consumption. Other nodes join a cluster based on the signal strength of each cluster head. If more than one cluster head has the same signal strength, the cluster head with a low ID will be selected. More details regarding the network construction will be omitted, as this is not the focus of this article.

The dataset is obtained from the TAO (Tropical Atmosphere Ocean) project [37] of NOAA (National Oceanic and Atmospheric Administration). The TAO project enabled real-time collection of high quality oceanographic and surface meteorological data for monitoring, forecasting and understanding of climate swings associated with El Nino and La Nina. More detail of the dataset will be given later.

There are three datasets with different distribution used here. The first one is a uniform distribution dataset generated by "unifrnd" function in Matlab ; the second one is a Poisson distribution dataset, generated by "poissrnd" function in Matlab (with Lambda = 200); and the last one is an actual dataset from wind direction of the TAO project. More detail of the real dataset is given in Table 6. It contains 2000 samples, selected from a continuous time interval (1992–1993) with invalid data removed. The measure range of wind direction is [0, 360); the resolution is 1.4. The accuracy is 5–7.8, which is much greater than the resolution. Here, we choose the highest accuracy, so $L=\frac{360-0}{5}=72$. For comparison, the other two datasets also have the same N and L, *i.e.*, N = 2000 and L = 72.

### 6.2. Analysis and Evaluations of MFSDA-I

Both theoretic analysis and experimental evaluation of MFSDA-I will be given in this section. As we discussed earlier, the concerned topic of RCDA is similar to the one of this paper, and EERCDA is an improve scheme of RCDA regarding the reduction of communication costs. Both of them will be chosen as candidates.

#### 6.2.1. Communication Cost of MFSDA-I

Communication cost can be measured by considering the package size. In the proposed scheme, data transmission can be divided into two categories.

One is intra-cluster data transmission, namely data transmission between *CM* and *CH*. The package size is $DL_{11}=\left|header|+|IDsrc|+|IDdest|+|timestamp|+|msgType|+\right|C_{11}\left|+|S\right|$.

The other is inter-cluster data transmission, namely, data transmission between *CH*s or data transmission between the *CH* and the server. The package size is $DL_{12}=\left|header|+|IDsrc|+|IDdest|+|timestamp|+|msgType|+\right|C_{12}\left|+|S\right|$.

The sensing data do not need to be coded in intra-cluster data transmission; thus, the length of plaintext corresponding to $C_{11}$ is $\left\lceil log_{2}L\right\rceil$.

Data will be encoded before inter-cluster data transmission. Because the number of elements in the encoded vector is L and the length of each element is at least $\left\lceil log_{2}N\right\rceil$, so the total plaintext length is $L\left\lceil log_{2}N\right\rceil$.

Let us assume that the ratio of the ciphertext and plaintext length in both stages is linear and the ratio $\alpha_{1}$ and $\alpha_{2}$, respectively. Then:

$$\begin{array}{c} DL_{11}=k_{1}+\alpha_{1}\left\lceil log_{2}L\right\rceil \\ DL_{12}=k_{1}+\alpha_{2}L\left\lceil log_{2}N\right\rceil \end{array}$$

where $k_{1}=\left|header\right|+\left|IDsrc\right|+\left|IDdest\right|+\left|timestamp\right|+\left|msgType\right|+\left|S\right|$.

For a uniform distribution, the inter-cluster data transmission can be further reduced to:

$$DL_{12u}=k_{1}+\alpha_{2}L\left\lceil log_{2}\frac{N}{L}\right\rceil$$

#### 6.2.2. Communication Cost of RCDA

In the first step of RCDA-HETE, namely intra-cluster data transmission, the packet size is $DL_{21}=\left|header|+\right|C_{21}\left|+|S\right|=k_{2}+\left|C_{21}\right|$.

Note that, although the packet sizes of MFSDA, RCDA and EERCDA are different, because all of them use the same signature, |S| can still be the same, as long as the appropriate elliptic curve and parameters are chosen. If we choose the same test platform, the |header| will also be the same. The encryption algorithm is consistent with MFSDA in the same stage, so:

$$DL_{21}=k_{2}+\alpha_{1}\left\lceil log_{2}L\right\rceil$$

In the second stage of RCDA-HETE and all stages of RCDA-HOMO, the packet size is $DL_{22}=k_{2}+\left|C_{22}\right|$.

The aggregation of sensing data is the concatenation of all messages from *CM*. The data length of each sensing datum is at least $\left\lceil log_{2}L\right\rceil$, so the total plaintext size is at least $N\lceil log_{2}L\rceil$; the encryption algorithm is the same as the one used in the second stage of MFSDA, so the packet size is:

$$DL_{22}=k_{2}+\alpha_{2}N\left\lceil log_{2}L\right\rceil$$

$\alpha_{2}>\alpha_{1}\geq 1$, so $DL_{22}>DL_{21}$, which means that RCDA-HETE is much more efficient than RCDA-HOMO in terms of communication cost.

#### 6.2.3. Communication Cost of EERCDA

To reduce the energy consumption of message transmission in RCDA [12] and to achieve more energy and bandwidth efficiency, EERCDA [13] uses a *differential data transfer method*. In EERCDA, the difference data, rather than raw data from the sensor node, are transmitted to the cluster head.

EE-RCDA includes two data transmission phase: *reference data transfer session* and *subsequent data transfer session*.

The first stage is the same as RCDA-HOMO. Each senor transmits raw data (reference data) to the server; thus, the packet size in this stage is still:

$$DL_{31}=k_{2}+\alpha_{2}N\left\lceil log_{2}L\right\rceil =DL_{22}$$

The differential data are transmitted to cluster head in the second stage. Then, the *server* can recover the raw data using reference data and differential data. The total packet size is $DL_{32}=\left|header|+\sum |ID|+\right|C_{32}\left|+|S\right|=k_{2}+\sum |ID|+|C_{32}|$.

Assume that the number of nodes whose data has changed is ęÂN at each query in this stages, then:

$$DL_{32}=k_{2}+\beta N\left\lceil \log N\right\rceil +\alpha_{2}\beta N\left\lceil \log L\right\rceil$$

Let us compare EERCDA with RCDA-HOMO first. EERCDA is much more efficient than RCDA-HOMO, if ${DL}_{32}<{DL}_{31}$, which means $\beta <\frac{\alpha_{2}\left\lceil log_{2}L\right\rceil }{\left\lceil log_{2}N\right\rceil +\alpha_{2}\left\lceil log_{2}L\right\rceil }$.

Now, let us compare EERCDA with RCDA-HETE. Because $DL_{22}=DL_{31}$, we only need to compare $DL_{21}$ to $DL_{32}$. In general, βN≫1, $\alpha_{1}<\alpha_{2}$, so: $k_{2}+\alpha_{1}\left\lceil log_{2}L\right\rceil \ll k_{2}+\alpha_{2}\beta N\left\lceil log_{2}L\right\rceil \ll k_{2}+\beta N\left\lceil log_{2}N\right\rceil +\alpha_{2}\beta N\left\lceil log_{2}L\right\rceil$.

That is to say, $DL_{21}\ll DL_{32}$, which also means EERCDA will consume more energy than RCDA-HETE.

#### 6.2.4. Evaluation of MFSDA-I

According to the analysis above, the communication cost of EERCDA is much greater than that of RCDA-HETE, while only in specific conditions is it much more efficient than RCDA-HOMO. To highlight the advantages of this scheme, we compare MFSDA to RCDA-HETE.

|IDsrc|+|IDdest|+|timestamp| in MFSDA is used to achieve a much higher security level. The bits will also be needed by RCDA and EERCDA, if they want to achieve similar security property. At the same time, the packet header may contain IDdest , timestamp, *etc.* in platforms, such as TinyOS 2.x. Therefore, it can be ignored during the comparison. |msgType| only need one bit, so can also be ignored. Therefore, there is no need to consider $k_{1}$ and $k_{2}$ during the comparison. Therefore, $DL_{21}\approx DL_{11}$, that is to say, MFSDA and RCDA-HETE have a similar communication cost in the first stage.

**Figure 3:**
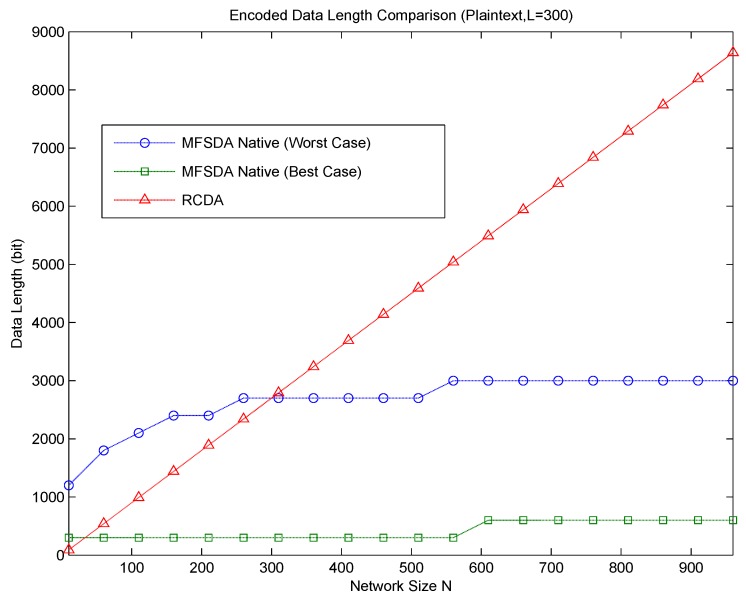
Communication cost comparison of RCDAand MFSDA.

When N>L, $DL_{12}-DL_{22}\approx \alpha_{2}\left(L\left\lceil log_{2}N\right\rceil -N\left\lceil log_{2}L\right\rceil \right)<0$. That is, MFSDA is more efficient than RCDA-HETE when N>L. Obviously, MFSDA is also more efficient than RCDA-HOMO and EERCDA in this condition. A case of communication cost comparison between RCDA and MFSDA is given in Figure 3.

Now, let us compare RCDA to MFSDA-I on wind direction, which is a real dataset obtained from the TAO project. According to the comparison result, MFSDA-II can reduce the communication cost dramatically at the cost of less accuracy loss. The measure rang TAO wind direction is [0,359], and the accuracy is five. When N = 100, the data length of RCDA is 700 bits, while the data length of MFSDA-I is 504 bits. The latter one is only 72% of the former one. When N = 300, the data length of RCDA and MFSDA-I are 2100 and 648, respectively. The data length of MFSDA-I has been reduced to 31% of RCDA. More detail is listed in Table 7.

### 6.3. Analysis and Evaluations of MFSDA-II

For MFSDA-II, we focus on the influence of the compression factor on communication cost and accuracy.

#### 6.3.1. Comparison with MFSDA-I

The data transmission of the intra-cluster, *i.e.*, data transmission from *CM* to *CH*, in the approximate scheme is the same as that in MFSDA-I. The compression step only has influence on inter-cluster data transmission, *i.e.*, data transmission between *CH*s or from *CH* to the *server*.

Because of the compression step, the item numbers of ${\vec{v}}_{k}$ and $\vec{V}$ are reduced from L to $L^{\prime }=\left\lceil \frac{L}{c}\right\rceil$, so the total data transmission will be reduced, as well. Then,

$$DL_{12}^{\prime }=k_{1}+\alpha_{2}L^{\prime }\times \left\lceil log_{2}N\right\rceil =k_{1}+\alpha_{2}\left\lceil \frac{L}{c}\right\rceil \left\lceil log_{2}N\right\rceil$$

For a uniform distribution, the result above can be further reduced to:

$$DL_{12u}^{\prime }=k_{1}+\alpha_{2}\left\lceil \frac{L}{c}\right\rceil \left\lceil log_{2}\frac{cN}{L}\right\rceil$$

**Figure 4:**
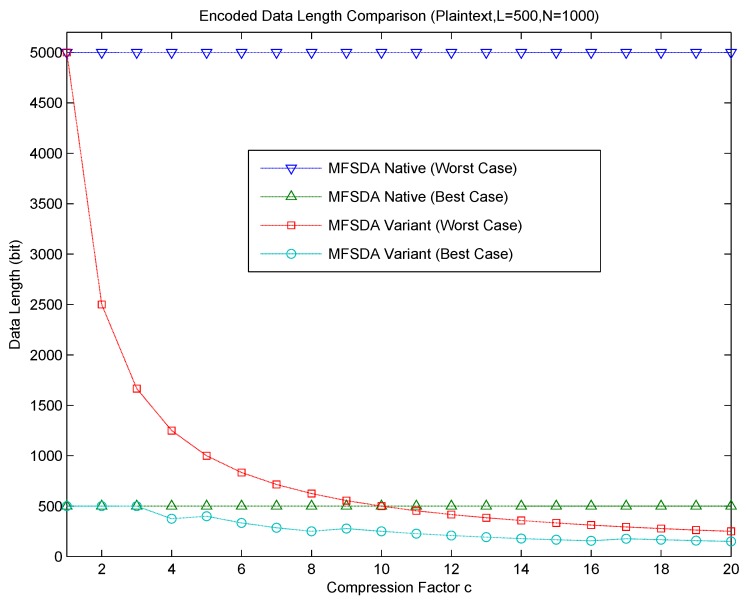
Communication cost comparison of MFSDA-I and MFSDA-II.

The relationship between communication cost and the compression factor is shown in Figure 4. The communication cost significantly decreases as the compression factor c increases in MFSDA-II. Error is introduced due to the loss of compression; accuracy analysis will be given next.

#### 6.3.2. Accuracy Analysis of MFSDA-II

Due to the use of the loss compression operation, this communication cost reduction will inevitably introduce errors. The final error may be modest due to the positive and negative errors existing simultaneously and offsetting each other.

The error rate of each statistic is in inverse proportion to or has a reverse trend with L and is proportional to or has a positive trend with c. For the same error rate request, the bigger the L, the bigger the value of maximum acceptable c. For the error rate of comparison-based statistics, such as ER(Error)MAX , ERMED and ERMIN, the reference boundaries are the determined ones, *i.e.*, the error rate is not beyond the borders. For the error rate of addition-based statistics, except for ERCNT, which is zero, the error rates of several other statistics constitute the reference boundary; the boundary is also used to describe the error variation trend and reference boundary range, and several specific case may out of the reference bounds. The reference boundaries of ERMAX, ERMED are $\pm \frac{c}{2L}$, *i.e.*, the boundary is directly related to ±c and shockingly enlarges as c increases. The upper bound of ERMIN, ERMAX and ERMED is the same. Due to rounding used in the compression step, ERMIN has a one-way increasing trend, and its lower bound is zero.

The CNT is the total active nodes number in the current query; thus, no error exists, namely ERCNT is zero. Therefore, the error reference bounds of ERMEAN and ERSUM are the same. Their lower bound is independent of c. Their upper bound is proportional to $c^{2}$ and is shockingly amplified as c increases.

The error reference bounds of ERVAR and ERSTD are also shockingly amplified as c increases, more so for ERVAR.

Compared to the comparison-based statistics, the coefficients of the addition-based statistics (such as ERVAR, ERMEAN, *etc.*) are $\frac{c}{L}$ times the former ones. c is much less than L; thus, $\frac{c}{L}$ is much smaller than one. Therefore, the error rate of comparison-based statistics is much greater than that of addition-based statistics. This is mainly because the addition-based statistics are calculated by using the data of all nodes, even though the data error of a single node is large; partial offset of the negative error and positive error exists simultaneously. While the comparison-based statistics are obtained from a single point of data, there is no such type of compensation.

#### 6.3.3. Evaluation of MFSDA-II

**Figure 5:**
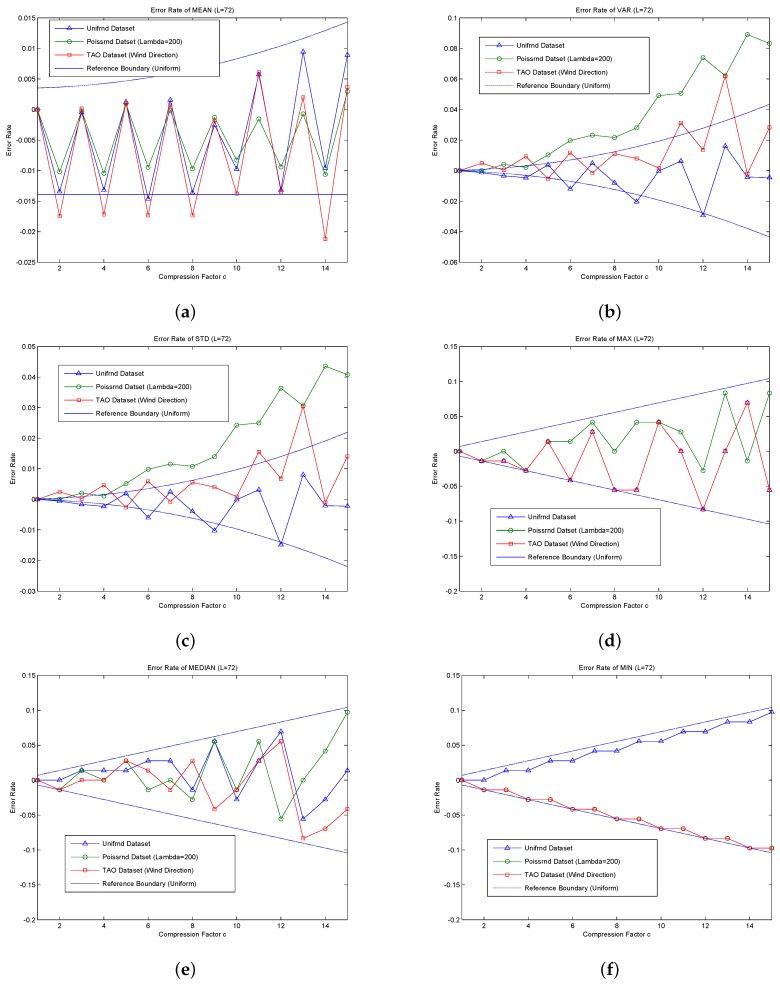
Accuracy evaluation of the MFSDA variant. (**a**) Error rate of MEAN; (**b**) error rate of VAR; (**c**) error rate of STD; (**d**) error rate of MAX; (**e**) error rate of MEDIAN; (**f**) error rate of MIN.

The statistical results obtained by MFSDA-I are accurate; thus, the accuracy evaluation is only performed on the MFSDA-II. The following experiments will analyze the error rate of each statistic as it changes with the increase of the compression factor. The results are listed in Figure 5.

Now, let us compared MFSDA-I with MFSDA-II based on the wind direction dataset of TAO, where the network size is N = 2000. The data length of MFSDA-I is 792 bits. When the compression factor is c = 2, the data length of MFSDA-II is 396 bits, which is only 50% of MFSDA-I. The maximum error rate of all statistical results is 2%, while the minimum error rate is 0.2%. When the compression factor is c = 4, the data length of MFSDA-II is 198 bits, which is only 25% of MFSDA-I. The maximum error rate of all statistical results is 2%, while the minimum error rate is 0.5%. More detail of the comparison result is listed in Table 8. More detailed accuracy analysis results of MFSDA-II will be given later.

The reference boundary is derived based on a uniform distribution. The more the actual data are similar to a uniform distribution, the higher the reference value that the boundaries will provide. As shown in Figure 5, the majority of error rates are in the reference boundary, though some statistical error rates are still beyond the reference boundary.

The majority of error rates of the *unifrnd dataset* are in the reference boundary. Compared to the other dataset, it can achieve higher accuracy (less error rate) for the same compression factor. The error rate reference formula of each statistic has a good prediction ability for the *unifrnd dataset*; thus, it can be used directly for compression factor choosing. For example, assuming that the accuracy requirement of ERSTD is approximately 2%, we can choose C = 15; the amount of data is compressed from L = 72 to $\frac{L}{c}=\frac{72}{15}=4.8$, which means that the final communication cost is only $\frac{1}{15}=6\%$ of that in MFSDA-I.

For the non-uniform distribution, some statistic error rates may be beyond the reference boundary. ERSTD and ERVAR of *poissrnd dataset* are beyond the reference boundary, so the reference error formula cannot be used directly. However, even in this condition, the error rate is still related to c. By the choice of a much less C, we can still greatly reduce the amount of communication cost and achieve high precision. For example, the ERVAR of *poissrnd dataset* reaches to 10% when c = 15. If we choose c = 10, the ERVAR will reduce to about 4%. The communication cost is reduced to $\frac{1}{10}=10\%$ of that in MFSDA-I.

As show in Figure 5, ERMAX, ERMIN and ERMED of all three datasets are in the reference boundary range; this is because the reference boundary of comparison-based statistics is the absolute boundary.

As the *TAO dataset of wind direction* is very similar to a uniform distribution, the greatest error rate is in the boundary range. In contrast, *poissrnd dataset* is less similar to a uniform distribution. If we calculate a suitable compression factor according the error formula, we can directly apply it to the *TAO dataset of wind direction*, and we may need to choose a much smaller c for the *poissrnd dataset*.

According the reference boundary formula, the error rate of each statistic is directly proportional to C and inversely proportional to L. That is to say, for the same accuracy requirement (error rate) of each statistic, the bigger the L, the largest the c. Due to the reduction of communication cost in MFSDA-II being $\frac{L}{L^{\prime }}=C$, therefore, the bigger the L, the more the energy saved.

For example, in the barometric pressure of the TAO project, $L=\frac{1100-800}{0.1}=3000$, still N = 2000. The experimental results show that, when c = 30, ERVAR is about 20%, and ERSTD is about 10%. The rest of the4 error rates are all less than 1%. L’ is reduced from 3000 to 100. Therefore, the amount of communication cost is only $\frac{1}{30}$ of MFSDA-I. Even if we choose a much smaller c = 20, to reduced ERVAR to 10%, the cost is still only $\frac{1}{20}$ of MFSDA-I.

## 7. Conclusions

This paper presents two cost-effective multi-functional secure data aggregation schemes (*i.e.*, MFSDA-I and MFSDA-II) for distributed systems, such as a mobile sensor network. Both schemes can provide *value-preserving* and *order-preserving* during in-network aggregation and, thus, obtain multiple statistics (both addition-based and comparison-based statistics) in a query. The *MFSDA-I* scheme can obtain accurate results, while the *MFSDA-II* scheme can obtain an approximate result with much less energy consumption. Analysis and evaluation results show that MFSDA scheme has great performance with respect to functionality, security and cost effectiveness.

## Figures and Tables

**Table 1:** Comparison of secure data aggregation (SDA) schemes on functionality. Y, yes; N, no; P, partly; MFSDA, multi-functional secure data aggregation.

	Summation-Based Statistics	Comparison-Based Statistics	Single Query
Castelluccia *et al.* [11]	Y	N	N
Roy *et al.* [9]	Y	N	N
Lin *et al.* [14]	Y	N	N
Li *et al.* [29]	Y	N	N
He *et al.* [30]	Y	N	N
Yang *et al.* [6]	Y	N	N
Lu *et al.* [28]	Y	N	N
Acharya *et al.* [24]	N	P	N
Ertaul *et al.* [26]	N	P	N
Samanthula *et al.* [27]	N	P	N
RCDA [12]	Y	Y	Y
MFSDA	Y	Y	Y

**Table 2:** Comparison of SDA schemes on adaptive dynamic networks.

	Not Support	Support, with Extra Cost	Support, without Extra Cost
Castelluccia *et al.* [11]	N	Y	N
Roy *et al.* [9]	N	Y	N
Lin *et al.* [14]	N	Y	N
Li *et al.* [29]	N	Y	N
He *et al.* [30]	Y	N	N
Yang *et al.* [6]	Y	N	N
Acharya *et al.* [24]	Y	N	N
Ertaul *et al.* [26]	Y	N	N
Samanthula *et al.* [27]	Y	N	N
RCDA [12]	N	N	Y
MFSDA	N	N	Y

**Table 3:** Basic notation.

Symbol	Description	Symbol	Description
$x_{k};X_{LB};X_{UB}$	Sensing data $x_{k}\in \left(X_{LB},X_{UB}\right]$	*a*	Accuracy requirement of $x_{k}$
*c*	Compressing factor	*L*	$L=\lceil \frac{X_{UB}-X_{LB}}{a}\rceil$
$y_{k}$	Mapping data, $y_{k}\in \left(0,L\right]$	*N*	Network size
$z_{k}$	Compression data of $y_{k}$	$f_{m};f_{m}^{-1}$	Mapping function and its inverse
${\vec{v}}_{k};{{\vec{v}}_{k}}^{\prime }$	Encoding data of $y_{k}$ or $z_{k}$	$f_{c};f_{c}^{-1}$	Compression function and its inverse
${\vec{V}}_{k};{{\vec{V}}_{k}}^{\prime }$	Aggregation of encoding data	$f_{e};f_{e}^{-1}$	Encoding function and its inverse
CH	cluster head	CM	cluster member

**Table 4:** Details of the values at each step in the example.

Sensor ID	Sensing Data	Mapping Data	Encoded Data
1	23	3	(0 0 1 0 0)
2	25	5	(0 0 0 0 1)
3	16	reject	reject
4	23	3	(0 0 1 0 0)
5	48	reject	reject
6	22	2	(0 1 0 0 0)
7	23	3	(0 0 1 0 0)
8	24	4	(0 0 0 1 0)

**Table 5:** Comparison of SDA schemes on security.

	A1	A2	A3	A4	B1	B2
Castelluccia *et al.* [11]	Y	N	N	N	Y	N
Roy *et al.* [9]	N	N	Y	N	Y	N
Lin *et al.* [14]	Y	N	N	N	Y	N
Li *et al.* [29]	N	N	N	N	Y	N
He *et al.* [30]	Y	N	N	N	N	N
Yang *et al.* [6]	Y	N	N	N	N	N
Acharya *et al.* [24]	Y	N	N	N	Y	N
Ertaul *et al.* [26]	Y	N	N	N	Y	N
Samanthula *et al.* [27]	Y	N	N	N	Y	N
RCDA [12]	Y	N	E	N	Y	N
MFSDA	Y	Y	Y	Y	Y	N

**Table 6:** Data description of the real dataset.

Data Name	Wind Direction
Data source	TAO (Tropical Atmosphere Ocean) [37] project of
	NOAA (National Oceanic and Atmospheric Administration)
Data range	[0,359]
Accuracy	5
L	72

**Table 7:** Data length comparison between MFSDA-I and RCDA based on WindDirectionof the TAO project (units: bits).

	N = 50	N = 100	N = 150	N = 200	N = 250	N = 300	N = 350	N = 400
**RCDA**	350	700	1050	1400	1750	2100	2450	2800
**MFSDA-I**	432	504	576	576	576	648	648	648

**Table 8:** Comparison between MFSDA-I and RCDA based on Wind Direction of TAO Project (units: bits).

	MFSDA-I	MFSDA-II						
	Data Length	Data Length	Accuracy					
			MEAN	VAR	STD	MAX	MIN	MEDIAN
c=2	792	396	-2%∼0.2%	±0.5%	±0.2%	±1%	±1%	±1%
c=3	792	264	-2%∼0.2%	±0.5%	±0.2%	±2%	±2%	±2%
c=4	792	198	-2%∼0.2%	±1%	±0.5%	±3%	±3%	±3%
c=5	792	158	-2%∼0.2%	±1%	±0.5%	±3%	±3%	±3%

## Appendix A. Homomorphic Encryption Scheme

The proposed scheme uses an EC-ElGamal-based homomorphic encryption algorithm derived from [32]. The algorithm consists of four procedures: setup, KeyGen, encryption and decryption. Details are illustrated in Algorithm A1.

**Algorithm 1:** Homomorphic encryption scheme.
1: **procedure**Setup ▹ Initialization 2: *p* is a prime number 3: *E* is an elliptic curve over a finite field $F_{p}$, 4: while *G* is the generator. 5: **return** para=<E,p,G> 6: **end** **procedure** 7: **procedure** KeyGen ▹ Key Generation 8: $KeyPriBS\in F_{p}$ 9: KeyPubBS←KeyPriBS×G 10: **return** key=<KeyPriBS,KeyPubBS> 11: **end** **procedure** 12: **procedure** HEnc(m,KeyPubBS) ▹ Encryption 13: M←map(m) 14: $R\leftarrow r\times G,where\;r\in_{R}F_{p}$ 15: S←M+r×KeyPubBS 16: **return** $C_{m}=<R,S>$ 17: **end** **procedure** 18: **procedure** HDec($C_{m},KeyPriBS$) ▹ Decryption 19: M←S-KeyPriBS×R 20: m←rmap(M) 21: **return** *m* 22: **end** **procedure**

**Theorem 1.** *(Homomorphic property) Algorithm A1 has an additive homomorphic property, namely the summation arithmetic in plaintext is equivalent to summation arithmetic in cipher text*.

$$HEnc\left(m_{1}+m_{2}\right)=HEnc\left(m_{1}\right)⨁HEnc\left(m_{2}\right)$$

**Proof**: $HEnc\left(m_{1}\right)⨁HEnc\left(m_{2}\right)=\left(R_{1},S_{1}\right)+\left(R_{2},S_{2}\right)=\left(R_{1}+R_{2},S_{1}+S_{2}\right)=\left(R_{sum},S_{sum}\right)$ $M_{sum}=S_{sum}-KeyPriBS\times R_{sum}=\left(S_{1}+S_{2}\right)-KeyPriBS\times \left(R_{1}+R_{2}\right)=S_{1}-KeyPriBS\times R_{1}+S_{2}-KeyPriBS\times R_{2}=\;M_{1}+M_{2}$ $HDec\left(HEnc\left(m_{1}\right)⨁HEnc\left(m_{2}\right)\right)=rmap\left(M_{sum}\right)=rmap\left(M_{1}+M_{2}\right)=rmap\left(M_{1}\right)+rmap\left(M_{2}\right)=\;m_{1}+m_{2}$ $HEnc\left(m_{1}+m_{2}\right)=HEnc\left(HDec\left(HEnc\left(m_{1}\right)⨁HEnc\left(m_{2}\right)\right)\right)=HEnc\left(m_{1}\right)⨁HEnc\left(m_{2}\right)$ End of proof.

## Appendix B. Identity-Based Signature Scheme

The identity-based signature (IBS) scheme is based on IBE. The users’ public identity information (ID, email, *etc.*) is used as the public key for signature verification in the IBS scheme, which can effectively solve the management problems in PKI public-key certificates.

The algorithm consists of four parts of setup, extract, signature and verify. BS using the master key MSKfor each node generates a signature private key based on their ID, the node using the private key for generating data of signature and data-receiving nodes using the sending node ID as the public key for the signature verification to verify the integrity of the information received.

## Appendix C. Theorem 2 and Its Proof

**Theorem 2.** *The homomorphic encryption used in the proposed scheme can ensure the vector obtained at the server after decryption is the aggregated result of all encoded data of the active nodes*.

**Algorithm 2:** Identity-based signature scheme.
1: **procedure**Setup ▹ Initialization 2: <E,p,G> is the same as in Algorithm A1 3: $msk\in_{R}F_{p}$ 4: P←msk×G 5: $H_{1},H_{2}$ are hash functions 6: **return** $para=<E,p,G,P,H_{1},H_{2}>$ 7: **end** **procedure** 8: **procedure** Extract($ID_{k}$) ▹ Key Generation 9: $R\leftarrow r\times G,where\;r\in_{R}F_{p}$ 10: $v\leftarrow r+H_{1}\left(ID_{k},R\right)\times msk$ 11: **return** $KeySign_{k}=<R,v>$ 12: **end** **procedure** 13: **procedure** Signature($msg,KeySign_{k}$) ▹ Signature 14: $T\leftarrow t\times G,where\;t\in_{R}F_{p}$ 15: $z\leftarrow t+H_{2}\left(ID_{k},msg,R,T\right)\times v$ 16: **return** $S_{k}=<R,T,z>$ 17: **end** **procedure** 18: **procedure**Verify($S_{k},msg,ID_{k}$) ▹ Verification 19: $h_{1}\leftarrow H_{1}\left(ID_{k},R\right)$ 20: $h_{2}\leftarrow H_{2}\left(ID_{k},msg,R,T\right)$ 21: A←z×v 22: $B\leftarrow T+h_{2}\times \left(R+h_{1}\times P\right)$ 23: **return** A==B 24: **end** **procedure**

**Proof**: Sensor data are encoded into ${\vec{v}}_{k}$ at the H-sensor and then are encrypted into ${\vec{c}}_{k}$ using the public key KeyPubBS. Aggregation at the intermediate node directly performs on the ciphertext ${\vec{c}}_{k}$; the final result obtained by the server is $\sum_{k=1}^{N}{\vec{c}}_{k}$.

$$\sum_{k=1}^{N}{\vec{c}}_{k}=\sum_{k=1}^{N}\left(HEnc\left({\vec{v}}_{k}\right)\right)$$

According to Theorem 1,

$$\sum_{k=1}^{N}\left(HEnc\left({\vec{v}}_{k}\right)\right)\Leftrightarrow HEnc\left(\sum_{k=1}^{N}{\vec{v}}_{k}\right)$$

so,

$$\sum_{k=1}^{N}{\vec{c}}_{k}=HEnc\left(\sum_{k=1}^{N}{\vec{v}}_{k}\right)$$

The final aggregation result $\vec{V}$ can be obtained from $\sum_{k=1}^{N}{\vec{c}}_{k}$ by decryption,

$$\vec{V}=HDec\left(\sum_{k=1}^{N}{\vec{c}}_{k}\right)=\sum_{k=1}^{N}{\vec{v}}_{k}$$

End of proof.

## Appendix D. Detail for the Example in Section 4.3.

The final aggregation result for example in Section 4.3 is $\vec{V}=\sum {\vec{v}}_{k}=\left(2\;1\;2\;1\;1\right)$. We can obtain each statistic from the final aggregated vector using the formulas provided in Section 4.2.

$$Cnt=\sum_{i=1}^{L^{\prime }}n_{i}=2+1+2+1+1=7$$

$$\begin{array}{cc} Sum(\hat{x}) & =\sum_{i=1}^{L^{\prime }}n_{i}f_{m}^{-1}\left(f_{c}^{-1}\left(i\right)\right)\\ & =2\times \left(\frac{4\times 1-2}{10}+9\right)+1\times \left(\frac{4\times 2-2}{10}+9\right)+2\times \left(\frac{4\times 3-2}{10}+9\right)\\ & \;\;\;\;+1\times \left(\frac{4\times 4-2}{10}+9\right)+1\times \left(\frac{4\times 5-2}{10}+9\right)\\ & =69.2 \end{array}$$

$$\begin{array}{cc} Sum({\hat{x}}^{2}) & =\sum_{i=1}^{L^{\prime }}n_{i}{\left(f_{m}^{-1}\left(f_{c}^{-1}\left(i\right)\right)\right)}^{2}\\ & =2\times {\left(\frac{4\times 1-2}{10}+9\right)}^{2}+1\times {\left(\frac{4\times 2-2}{10}+9\right)}^{2}+2\times {\left(\frac{4\times 3-2}{10}+9\right)}^{2}\\ & \;\;\;\;+1\times {\left(\frac{4\times 4-2}{10}+9\right)}^{2}+1\times {\left(\frac{4\times 5-2}{10}+9\right)}^{2}\\ & =686.2400 \end{array}$$

$$Avg=Sum(\hat{x})/Cnt=9.8857$$

$$E(\hat{x})=Avg=9.8857$$

$$E\left({\hat{x}}^{2}\right)=Sum\left({\hat{x}}^{2}\right)/Cnt=98.0343$$

$$Var\left(\hat{x}\right)=E\left({\hat{x}}^{2}\right)-E{\left(\hat{x}\right)}^{2}=0.3072$$

$$STD=\sqrt{VAR(\hat{x})}=\sqrt{0.3072}=0.5543$$

$$Max=f_{m}^{-1}\left(f_{c}^{-1}\left(i_{\max}\right)\right)=\frac{4\times 5-2}{10}+9=10.8$$

$$Min=f_{m}^{-1}\left(f_{c}^{-1}\left(i_{\min}\right)\right)=\frac{4\times 1-2}{10}+9=9.2$$
